# Am I getting an accurate picture: a tool to assess clinical handover in remote settings?

**DOI:** 10.1186/s12909-017-1067-0

**Published:** 2017-11-15

**Authors:** Malcolm Moore, Chris Roberts, Jonathan Newbury, Jim Crossley

**Affiliations:** 1 Rural Clinical School, Australian National University Medical School, 54 Mills Rd, Acton, ACT 2601 Australia; 2 Northern Clinical School, Sydney Medical School, University of Sydney, Sydney, Australia; 3 Rural Clinical School, University of Adelaide, Adelaide, Australia; 4 Medical School, University of Sheffield, Sheffield, UK; 5 Broken Hill University Department of Rural Health, University of Sydney, Broken Hill, Australia

**Keywords:** Clinical handover, Work-based assessment, Communication skills, Medical education

## Abstract

**Background:**

Good clinical handover is critical to safe medical care. Little research has investigated handover in rural settings. In a remote setting where nurses and medical students give telephone handover to an aeromedical retrieval service, we developed a tool by which the receiving clinician might assess the handover; and investigated factors impacting on the reliability and validity of that assessment.

**Methods:**

Researchers consulted with clinicians to develop an assessment tool, based on the ISBAR handover framework, combining validity evidence and the existing literature. The tool was applied ‘live’ by receiving clinicians and from recorded handovers by academic assessors. The tool’s performance was analysed using generalisability theory. Receiving clinicians and assessors provided feedback.

**Results:**

Reliability for assessing a call was good (G = 0.73 with 4 assessments). The scale had a single factor structure with good internal consistency (Cronbach’s alpha = 0.8). The group mean for the global score for nurses and students was 2.30 (SD 0.85) out of a maximum 3.0, with no difference between these sub-groups.

**Conclusions:**

We have developed and evaluated a tool to assess high-stakes handover in a remote setting. It showed good reliability and was easy for working clinicians to use. Further investigation and use is warranted beyond this setting.

**Electronic supplementary material:**

The online version of this article (10.1186/s12909-017-1067-0) contains supplementary material, which is available to authorized users.

## Background

### The importance of handover

The provision of good clinical handover has been identified as a vital part of safe, effective medical care [1–3]. Handover has been studied in hospital and pre-hospital settings. Most research has focussed on recording whether important categories of information have been communicated; however, other elements have been studied. These include the behaviour of handover teams and the communication characteristics of both parties involved in the handover [4, 5]. What has not been addressed is empirical evidence for the measurement of the quality of clinical handover within rural and remote healthcare settings. In addition, although handover skills are increasingly taught to medical students [6], there is little research on students giving handover in clinical settings. This paper is designed to address that gap by describing the development and evaluation of a handover tool in a critical setting where remote clinic staff and students are required to hand over to a general practitioner by telephone.

### Models of handover

Handover has been defined as ‘the exchange between health professionals of information about a patient accompanying either a transfer of control over, or of responsibility for, the patient’ [7]. A review of handover tools broadened this definition to include seven ‘framings’ that capture the primary purposes of handover [8]. Three of these relate directly to the handover conversation: ‘information processing’ – the most closely studied element of handover; the identification of a ‘stereotypical narrative’ (and deviations from this); and the ‘resilience’ of the handover in revealing errors in its assumptions. Four other framings relate to the broader context of handover and subsequent actions: ‘accountability’ for tasks; ‘social interaction’ in a handover group; ‘distributed cognition’ – networking with relevant people; and ‘cultural norms’ within the organisation.

A variety of handover formats is used including mnemonic-based frameworks, paper forms and electronic tools. The use of a standardised format has been shown to improve outcomes of handover – such as information transfer – but evidence of improved patient outcomes is scant [4]. The ISBAR mnemonic describes a structured form of handover that is used widely and has been endorsed by the World Health Organisation [9]. (Table 1) It was adapted from a structure used in the armed forces and the aviation industry on the assumption that standardisation would improve outcomes – however this has not been reliably shown to date in the healthcare setting [7]. ISBAR has been shown to improve the content and clarity of inter-professional non face-to-face handover in hospital settings [10] and is suited to a range of clinical contexts. The use of ISBAR as the basis for a handover conversation addresses the three relevant ‘framings’ described above: it triggers the clinician handing over to provide key information; and, in articulating a clinical assessment, promotes the consideration of recognisable clinical patterns and sources of error. Because ISBAR doesn’t specify categories of information relevant to specific settings – such as a surgical ward handover – it can be used in hospital and community settings.

**Table 1:** The ISBAR handover framework

**I**ntroduction – identify yourself, your role and the patient **S**ituation – state the patient’s main problem **B**ackground – give the relevant clinical history **A**ssessment – give the relevant observations and assessment of the patient’s condition **R**ecommendation – state the course of action or response that you are recommending	

### The assessment of handover

The literature describes a range of assessment tools that have been used to assess clinical handovers. Most hospital-based studies use tools that record how many specific pieces of information are handed over against an agreed list, including items included in frameworks such as ISBAR [10–15]. Other studies use a checklist to measure items related to effective clinical reasoning [16] or analyse patterns of handover communication [17]. Broader elements of handover assessment are identified in a literature review of pre-hospital handover research: clear speech; active listening; structure; amount of feedback given; and documentation [18]. Few studies were identified using the receiving clinician as the handover assessor. Two recently developed handover assessment tools are described below.

The Handoff CEX (clinical evaluation exercise) is a handover assessment tool developed to assess handover between teams at the change of shift in a general hospital [19]. It asks assessors to score six assessment domains and a global rating using a nine-point Likert scale. The rubric specifies elements of multi-patient handover and requires judgement of each domain across the whole handover. The Verbal Handoff Assessment Tool (VHAT) was developed for a paediatric hospital setting, using the I-PASS handover framework [20]. It scores 11 items, on a rubric from 0 to 3, using audio recordings.

These tools both assess items beyond simple information transfer and seek to place the handover into a specific context of hospital ward handover. They consider elements of communication, teamwork and clinical judgement that are part of the broader ‘framings’ of handover. The Handoff CEX can potentially be used by working clinicians. However, the research shows difference in scores between clinicians and independent observers which could indicate that is difficult for clinicians – particularly peers - to make complex judgements across these six domains. The VHAT performed well as an assessment tool but has not been reported as used by working clinicians.

### The local context

Broken Hill is an outer regional centre of 18,500 people in Far West New South Wales. The region is socioeconomically disadvantaged with a high burden of chronic disease, and increased prevalence of behavioural risk factors such as smoking in pregnancy, obesity, and physical inactivity [21]. These issues are amplified across Indigenous communities in the region. As with other rural and remote centres, the Broken Hill health workforce includes a significant fly-in-fly-out population. The Broken Hill medical student program has been described in detail elsewhere [22]. It includes senior medical students on longitudinal integrated placement of up to a full year and students on 2 to 4 week placements. Clinical learning occurs in community and hospital settings, including remote healthcare teams and with the Royal Flying Doctor Service (RFDS). The RFDS doctors provide a general practice as well as an aeromedical retrieval service to remote communities in many areas of Australia.

In locations where there is no resident doctor, telephone handover is given to RFDS doctors by resident remote nurses and medical students. It is critically important in this context that handover clearly communicates the patient’s condition. Callers are expected to give a structured handover that leads to an agreed understanding of the clinical situation and the required management. Some or all of that management will be provided on site by the caller and might include preparing for the RFDS to retrieve the patient to a larger centre. Medical students are supervised on site by registered nurses who liaise by telephone with the RFDS doctors providing regular clinics in these sites. The students are briefed, supported and debriefed by medical academics in Broken Hill, collaborating with the RFDS and the supervising nurses.

In this rural and remote context, our primary research question was ‘what factors impact on the reliability and validity of the assessment of telephone handovers from medical students and remote nurses to primary care doctors?’

## Methods

This was a cross-sectional, mixed methods study using both quantitative and qualitative measures to evaluate our assessment tool. We used the framework described by Fetters et al. in employing a convergent design that merged the data for interpretation and reporting [23]. In gathering validity evidence we used the approach of Downing – testing multiple sources of evidence for construct validity [24]. Qualitative data was analysed thematically to explore the extent to which the handover tool covers the relevant content domain and whether it is at the right level of cognitive complexity [25] and whether it provided meaningful scores. Reliability was assessed using generalisability theory [26]. We investigated whether there was a difference between scores of nurses and students and between students early and late in their placement.

### Constructing tool items

In order to develop a tool that was suited to the local context and enabled clinicians to assess the handover in a way that matched their concept of an effective handover, we used the method of Crossley and Jolly to ensure that the response scale should focus on competencies that are central to the activity observed [27]. In developing the content validity of the tool, one of the authors (MM) met separately with three focus groups of five registered nurses, seven RFDS doctors, and ten medical students on placement. Participants were asked about their understanding and experience of handover and the method that they used. These discussions revealed that the ISBAR framework for handover was widely taught and understood by students and nurses, and the RFDS doctors felt that it contained the elements that they wanted to hear in a handover. ISBAR covers the three important framings of the handover conversation, noted above, providing further evidence of content validity [8]. Choosing a small number of items avoided a long ‘tick-box’ approach which has been noted as a problem in work-based assessment [27]. These are high-stakes handovers with limited margin for uncertainty; ‘patient safety’ is the overarching frame of reference. Relevance and clarity of each of the items was assessed by the authors and further refined.

This draft of the Clinical Handover Assessment Tool (CHAT) was piloted by six of the RFDS doctors who had assisted in its development, in their routine work over 1 month. The first author met with this group to discuss the strengths and weaknesses of each item and to suggest modifications.

**Fig. 1:**
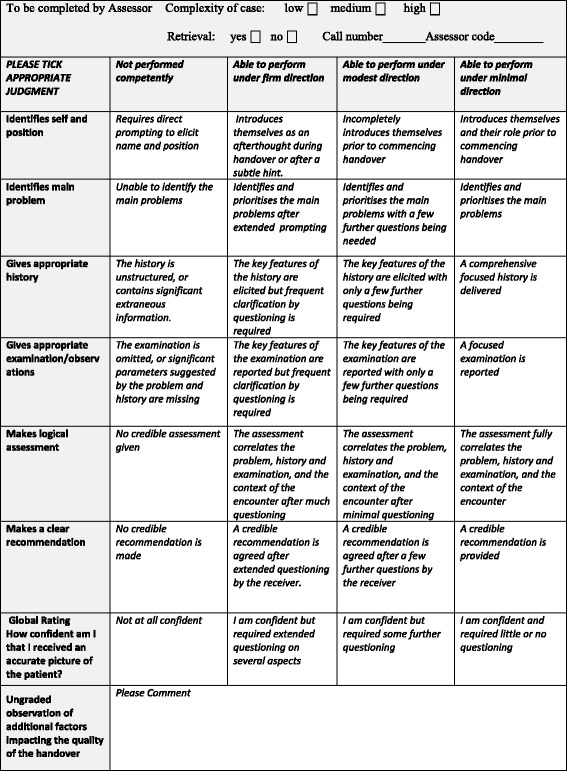
The Clinical Handover Assessment Tool

The tool was modified accordingly in the light of the work-based assessment literature [27]. The first item – ‘Introduction’ – was made more flexible to accommodate varying clinical situations. A clinical reasoning item – ‘Makes logical assessment’ – was added. The global assessment description was modified to ask, ‘How confident am I that I received an accurate picture of the patient from this handover?’ The assessment scale for each item was changed to use a rubric based on the amount of questioning or direction required for the caller to provide the desired information. This was based on evidence showing that aligning scales with the priorities of clinician assessors ‘increased assessor discrimination and reduced assessor disagreement’ [27]. Each item was scored on a Likert scale of 0–3. 0 = Not performed competently, 1 = Able to perform under firm direction, 2 = Able to perform under modest direction, and 3 = Able to perform under minimal direction. (Fig. 1)

The tool also collected data on the complexity of cases, as well as details of the caller, receiver, location and time of the call. Handover complexity was rated by the receiving doctor as low, medium or high. We hypothesized that calls of lower complexity would receive higher scores due to the relatively simple handover required.

Assessor training for use of the CHAT was provided to five academics from the research group using recorded handovers supplied by the RFDS. RFDS doctors, who had participated in the development of the tool, were also trained in its use.

As assessees, students had received varying amounts of training in the use of ISBAR from their home university. Most also received brief simulation training in Broken Hill although some students who went directly from their urban placement to the remote site did not. All nurses had received standardised training in ISBAR from the Health Service.

### Data collection

All students and nurses in the study sites were invited to participate prior to recording. We planned to sample remote telephone clinical handover (RTCH) recordings over 1 year for assessment by the academic group: 40 recordings of medical students (two per student from 20 students); and 48 recordings of remote nurses (four per nurse from 12 nurses). These calls were identified from call logs kept by students and nurses. The date and time of each call was matched with recordings made by the RFDS who supplied them to the research group. Recordings were checked to ensure that they were of a clinical handover. Where there were more than two calls recorded for an individual the earliest and latest available calls were included in the sample.

The sample of handover calls was allocated to members of the assessor group so that all calls had at least two assessors and each assessor received 25 calls. Where a call had been assessed by an RFDS doctor this was used as the second assessment, paired with an assessment by an academic. Assessments by academics and RFDS doctors were collated and entered into the study for analysis.

Qualitative data on the use of the tool were obtained by inviting RFDS doctors to complete a semi-structured survey by phone, discussing their use of the CHAT tool in the context of the usual practice in clinical handover to determine both intended and unintended consequences of the assessment (see Additional file 1). The survey asked questions about the tool’s appropriateness and ease of use as well as any suggested modifications. Academic assessors gave feedback on the CHAT tool individually and provided comments over five meetings of the research group.

### Data analysis

Descriptive and inferential statistics were generated. A Mann–Whitney U test was used to compare scores of handovers by students and nurses, handovers given early or late in a student’s placement and handovers of different complexity. The internal consistency of the total scale was measured using Cronbach’s alpha. The performance of the scale was analysed using generalizability theory, in order that future modifications of the handover tool can be planned that address the main sources of error identified in this initial study. In Generalisability theory the G–study quantifies the sources of potential error in the assessment simultaneously, using all of the available data [26]. A variance components analysis, within the General Linear Model, estimated the contribution that the wanted factor (the quality of the call) and the unwanted factors (e.g. the stringency or leniency of the assessor) made to the variation in handover assessment scores. Variance estimates were then combined using a spreadsheet to provide an index of reliability (the G coefficient) [28]. The overall checklist score was used as the dependent variable because factor analysis demonstrated a unitary structure and the items had been chosen to provide an exhaustive representation (rather than a sample of elements) of a ‘complete’ clinical handover in the rural and remote context. The G-study was based on a partially-nested model [29] with students/nurses as assessees and RFDS doctors and academics as assessors. D-studies were conducted to model the reliability of the tool with different numbers of assessors.

The qualitative data obtained from semi-structured interviews underwent thematic analysis by two researchers individually and collaboratively until consensus was reached [30].

Ethical approval for the study was obtained from the University of Sydney HREC and the Greater Western Area Health Service Ethics Committee.

## Results

### CHAT descriptive data

Ten of the 12 eligible nurses and 15 of 20 medical students consented to participate and had their calls to the RFDS recorded. All eight RFDS doctors consented to having their ratings of calls and focus group data included in the study. There were 55 calls, which were suitable for assessment – 37 by medical students and 18 by nurses - and 132 assessments were made on these calls. Students who had more than two recordings collected had three calls included for analysis. Calls were excluded if the assessor panel considered the calls did not constitute handover or if there were three or more missing data items.

The group mean for the checklist sub-total (6 items – maximum score 18) was 13.18 (SD 3.88). The item ‘Identifies main problem’ was scored highest - mean 2.50 (0.75) – and ‘makes a clear recommendation’ was lowest – mean 2.08 (0.97). The group mean for the global score (maximum score 3) was 2.30 (0.85). Scores are displayed in Table 2.

**Table 2:** CHAT mean scores for assessments of calls by checklist item, total scale score and global score

	N^a^	Minimum	Maximum	Mean (SD)
Introduction	129	0	3	2.42 (0.66)
Main problem	131	0	3	2.50 (0.75)
History	128	0	3	2.27 (0.81)
Examination	124	0	3	2.24 (0.81)
Logical assessment	128	0	3	2.14 (0.89)
Recommendation	127	0	3	2.08 (0.97)
Sub-total	132	0	18	13.18 (3.88)
Global score	132	0	3	2.30 (0.85)

^a^There were some missing data for sub-score items

Medical students and nurses showed no significant difference in the means of sub-total scores and global scores. There was no significant difference in scores of medical students making calls early or late in their clinical placement.

The complexity of handover cases was rated as: 24 low; 24 medium; 2 high; and 5 not rated. Low complexity cases - sub-total mean 14.60 (2.83)) were scored more highly than medium complexity cases - 12.66 (2.86) (*p* = 0.003).

### Scale analysis

The scale had a single factor structure with only ‘introduction’ being weakly loaded (0.33). The internal consistency of the scale items, calculated using Cronbach’s alpha, was 0.8.

The G-study of the reliability of assessing an individual call using the checklist sub-total showed 40% of variance related to call quality, 37% from call by assessor interaction - which reflects the consistent differences in stringency or leniency of assessors with calls - and 22% from the assessor - reflecting their stringency/leniency. The D-study gave a G-coefficient of 0.73 with four assessors, increasing to 0.80 with six assessors. The reliability of the global score alone was slightly lower (G = 0.70 with 6 assessors). (Table 3) The G-study of the reliability of assessing an individual caller was more speculative because most callers were only assessed on 2–3 calls. This was not enough to give a reliable estimate of the tool as an assessment of caller ability.

**Table 3:** Variance (G study) and reliability (D study) estimates) for assessing a call

Mean checklist scores			Mean global scores		
Variance estimates (G study)					
Variance Component	Estimate	Proportion	Variance Component	Estimate	Proportion
Call	0.159	40%	Call	0.197	28%
Assessor	0.088	22%	Assessor	0.147	21%
Call by Assessor	0.146	37%	Call by Assessor	0.352	51%
Residual	0.000	0%	Residual	0.000	0%
Reliability estimates (D study)					
Number of assessors	G nested	G crossed	Number of assessors	G nested	G crossed
1	0.40	0.52	1	0.28	0.36
2	0.58	0.69	2	0.44	0.53
3	0.67	0.77	3	0.54	0.63
4	0.73	0.81	4	0.61	0.69
5	0.77	0.84	5	0.66	0.74
6	0.80	0.87	6	0.70	0.77
7	0.83	0.88	7	0.73	0.80
8	0.84	0.90	8	0.76	0.82

### Assessor feedback

Four main themes emerged from the qualitative data: ease of use; appropriateness; specific context issues; and variability of the receiver’s communication style.

The RFDS receiving doctors and academic assessor panel all reported the tool was easy to use, with descriptions such as ‘user friendly’; ‘very easy to use…broke it up into easy sections’ and ‘quite straightforward’. The clinicians found it was sometimes difficult to fit into the workflow: ‘the form is fine [but] it’s another thing that we have to do’. One doctor reported it was ‘harder when the person (patient) wasn’t…such an easy consult’. The challenge was that ‘[the] main thing is to pick it up and use it.’

There was also consensus that the tool items were appropriate as a measure of handover quality. The clinicians supported ISBAR as a structure and that the tool reflected this. One clinician reported that whilst it separated those not performing competently it was ‘hard to differentiate the good from the great’.

There were issues for some items that related to the particular context of the calls. The introduction item was not always relevant, if the caller was known to the receiver, or if the receiver was returning a missed call. Examination findings were not always an important element of calls.

Several academic clinical assessors reported difficulty in making a judgement because of some variation in the RFDS receiving doctor’s communication style: some started asking questions early in the call, particularly if they were short of time. This was said to make assessing some calls difficult using the rubric (which is based on the amount of questioning needed). Some receivers didn’t seek a recommendation from a caller if they hesitated, rather proposing a management plan themselves. None of the RFDS doctors reported this as a difficulty while assessing calls ‘live’.

There were no reports of any unintended consequences with the use of the tool.

## Discussion

Our findings show that the CHAT has good reliability for assessing the quality of a high-stakes handover call. Assessors found it reasonably easy to use – even in the ‘live’ context – and felt that it assessed the handover in a way that matched their concept of the handover’s effectiveness. Evidence for its content validity was provided by the active involvement of clinician-assessors from the early stage of the research. The ISBAR framework was a logical choice in this context where it is widely taught and used. Clinician- and academic-assessors agreed that the items derived from this framework contained the essential elements of handover and this was confirmed by reference to the handover literature. The process of piloting and refinement underlined this.

Some construct validity evidence is provided by the difference in scores between low- and medium-complexity cases. Our hypothesis that low-complexity cases would receive higher scores was supported, the mean score being 15.3% higher than for high-complexity cases.

The G-coefficient of 0.80 with six assessors, using the checklist sub-total score, meets the generally accepted level of suitability for high-stakes judgements [26]. The G-coefficient of 0.73 with four assessors is consistent with use in formative assessment.

We have not presented the G-study data of the reliability of assessing individual callers in the results section because of the small number of calls per assessee. However, there is an interesting difference in the results of the checklist sub-total and of the global score. The checklist outperforms the global score as a measure of individual call quality but the global score outperforms the checklist as a measure of caller ability. This suggests that assessors may be making a broader assessment of the caller’s competence (global score) over and above their performance on individual items in a call, but more data are required to draw firm conclusions over this.

The CHAT was found to be a ‘user-friendly’ tool, suitable for use in routine clinical work. The familiarity of clinician-assessors with ISBAR is likely to have contributed to the tool’s high level of acceptance, aligning with their existing handover processes. It also contains a small number of items for scoring - similar to the Handoff CEX which is a hospital-specific tool.

Whilst there are many handover frameworks in use internationally, most are used in-hospital and are structured to ensure that locally specific information is communicated (I-PASS, for example [20]). The CHAT provides an assessment of quality that encompasses a shared understanding of clinical assessment and treatment priorities. We have only provided data evaluating CHAT in the remote high-stakes setting, but there is no reason in principle why it should not be equally well-suited to a range of in- and out-of-hospital settings where single-patient high-stakes handovers require an assessment and recommendation to be made. This includes many acute handover calls from junior to more senior clinical staff. It is potentially more flexible than other tools developed in recent years, that share similarities with the CHAT, but are designed for in-hospital use and multi-patient handover.

Some issues have been identified that could be addressed by further modifying the tool. Assessors found that the ‘Introduction’ item was often hard to rate because the caller and patient details were already known and left unsaid. This makes sense in the current study setting but would need to be considered in other contexts where this information is not known. Factor analysis showed that this item was weakly loaded and, therefore, could be omitted. Structurally, this would align with the SBAR mnemonic, also widely used in- and out-of-hospital [31].

Another issue related to the rubric’s use of the ‘amount of questioning required’ as an indicator of call quality. This measure was conceptualised as applying to the caller’s initial presentation of the case. It was assumed that further discussion would follow but that this would be less critical if the initial presentation was of high quality. This is consistent with the ‘two-way’ nature of handover, previously mentioned [1, 32]. Feedback from academic assessors indicated that the rubric could be problematic when the receiver has a more ‘interventionist’ style of conversation. This is a potential issue where assessments are being made from recordings but less so when the tool is being used by the clinician receiving handover. RFDS assessors perceived that they interrupted the caller earlier when they sensed the handover was of poorer quality. This issue underlines the importance of adequate training of clinicians using the tool.

### Strengths and limitations

This is the first assessment tool to be developed for the purpose of clinical handover in rural and remote settings. We acknowledge that this study was constrained by the limited number of calls per assessee. This related to several logistical problems in the identification and recording process. Our sampling pattern prevented us from estimating reliability for assessing the competence of callers over multiple calls. This also restricted the power of the study to find score variations attributable to experience and training.

The study was conducted in a remote location using telephone handover and the generalisability of the findings to other settings cannot be assumed. The researchers considered the possibility of linking the handover assessment to clinical outcomes: did the handover enable safe, appropriate ongoing care? It was not possible in the context of this study to link handover calls to outcomes due to the difficulty of tracking patient records through a system of multiple providers. This prevented us from matching the clinicians’ assessment of handover quality with an objective assessment of the patients’ condition.

### Future directions

The tool shows promise in the formative and progressive summative assessment of students and clinicians making individual handover calls and could be considered for further use and evaluation in that context. It can also be considered for use in other rural and remote sites where students and junior doctors are playing an active role in healthcare teams and demonstrating safe practice is important.

A larger study will be extremely valuable to determine the reliability of the tool for use in rating individual callers. This will help to clarify its suitability for use as a professional assessment tool [26].

## Conclusions

We have developed an assessment tool based on a structured handover format (ISBAR) that enabled clinicians to assess the handover in a way that matched their concept of an effective handover. We have provided some evidence for the validity of the tool by evaluating aspects of construct validity and generalisability. A larger study is required to assess the CHAT’s reliability in professional assessment. It could be considered for use in many contexts where single-patient handovers are made.

## Additional file

Additional file 1: Semi-structured interview: RFDS doctors. Question list. (DOCX 14 kb)

## Abbreviations

CHAT: Clinical Handover Assessment Tool
I-PASS: Illness severity, patient summary, action list, situation awareness, contingency planning, synthesis
ISBAR: Introduction, Situation, Background, Assessment, Recommendation
PACE: Patient, Assessment, Continuing, Evaluation
RFDS: Royal Flying Doctor Service
RTCH: Remote telephone clinical handover
VHAT: Verbal Handoff Assessment Tool
