# Supplementary material for: Am I getting an accurate picture: a tool to assess clinical handover in remote settings?
Source: BMC Med Educ. 2017 Nov 15;17:213. doi: 10.1186/s12909-017-1067-0 (PMC5688655; doi:10.1186/s12909-017-1067-0)
Supplement: Additional file 1: — Semi-structured interview: RFDS doctors. Question list. (DOCX 14 kb) [file 12909_2017_1067_MOESM1_ESM.docx]

**Semi-structured interview: RFDS doctors**

1. Overall, how do you feel about the quality of remote telephone clinical handovers?

2. Please identify the main difficulties you have found in receiving remote telephone clinical handovers.

3. What, if anything, would you change about the process of remote telephone clinical handovers?

4. Do you have any suggestions for further education to improve remote telephone clinical handovers? Please outline.

5. Do you think ISBAR is the appropriate framework for remote telephone clinical handovers? Please give reasons.

6. You have been using a form to make an assessment of handovers.

Please discuss how you have found the form regarding the following: ease of use; its appropriateness to assessing handover quality; and its use as an aid to giving feedback to the caller.

7. How do you manage a bad handover call?

8. Do you have any clarifications or further comments about the use of the form?
